# Normal and unusual days for dietary intake during the 12 months after a breast cancer diagnosis in women

**DOI:** 10.1007/s00394-022-02925-9

**Published:** 2022-06-24

**Authors:** Sonja H. Brunvoll, Vidar G. Flote, Eline H. Halset, Gro F. Bertheussen, Helle Skjerven, Jon Lømo, Inger Thune, Anette Hjartåker

**Affiliations:** 1 Department of Nutrition, University of Oslo, Oslo, Norway; 2 Department of Oncology, Oslo University Hospital, Oslo, Norway; 3 Department of Physical Medicine and Rehabilitation, St Olav University Hospital, Trondheim, Norway; 4 Department of Neuromedicine and Movement Science, Faculty of Medicine and Health Sciences, Norwegian University of Science and Technology, Trondheim, Norway; 5 Department of Research, Vestre Viken Hospital Trust, Drammen, Norway; 6 Department of Pathology, Oslo University Hospital, Oslo, Norway; 7 Department of Clinical Medicine, Faculty of Health Sciences, UiT, The Arctic University of Norway, Tromsö, Norway

**Keywords:** Atypical dietary days, Breast cancer, During adjuvant treatment, Free text fields, Pre-coded food diary, Unusual dietary days

## Abstract

**Purpose:**

There are several reasons to report days as being unusual with regard to dietary intake, including special occasions and celebrations. For breast cancer patients during the 12 month post-surgery period, unusual days may also include days that are affected by being a cancer patient. The aim of this study was to study dietary intake on “normal” and “unusual” days, and to study what is reported in “free text fields” of a food diary.

**Methods:**

Women (*n* = 456), mean age 55.5 years newly diagnosed with invasive breast cancer (stage I/II) were included in this clinical study. “Normal” and “unusual” days in general, over time and during the week and weekends were studied using repeated administration of a 7-day pre-coded food diary.

**Results:**

The breast cancer patients reported 26% of all days as unusual. The intake of energy, most nutrients, especially alcohol and sugar, red and processed meat, and sweets, cakes, and snacks was 5–126% higher, whereas intake of fiber, fruit and berries, vegetables, and dairy products was 7–17% lower on unusual than on normal days (*P* < 0.001). The same pattern was seen for normal/unusual days during the weekdays, weekends and over time. Finally, 99% of the breast cancer patients used the free text fields to report additional intake with a mean energy of 1.1 MJ/day.

**Conclusion:**

For breast cancer patients during the 12-month post-surgery period, unusual days are important drivers of total intake, especially for alcohol. The free text fields in the pre-coded food diary contributed substantially to the total intake.

## Introduction

To provide a measure of the usual dietary intake for a population, it is possible to use a dietary assessment method that asks about the usual diet (e.g. a food frequency questionnaire), or repeat administrations of a method that measures the daily dietary intake (e.g. 24-h recalls or food records/food diaries) [1, 2]. For the latter, the higher the number of repeat administrations, the more likely it represents the usual diet [2–4]. Food records usually span 3–7 days, while 2 non-consecutive days have been reported as sufficient for 24-h recalls, representing the usual diet for a population. Overall, the sampling by both methods should include weekdays and weekend days [3–6]. The dietary intake has been reported to fluctuate over the week, with typically a higher intake of energy, especially from alcohol, as well as a lower quality of the diet at the weekends compared with the weekdays [5, 7, 8]. A question about whether the day assessed is considered by the respondent as “normal” or “unusual” concerning food and drink consumption has been part of a Norwegian pre-coded food diary since it was developed for a nationwide study in 2000 [9, 10] and later used in several studies [11–17]. What an “unusual” day represents has, to our knowledge, not been previously examined in detail, and knowledge is scarce on how similar questions are answered and how such days are described and what they reflect [18]. However, one study from the US investigated food records with at least one atypical day, characterized as “more than usual” or “less than usual”, compared with food records without atypical days. The authors concluded that atypical days have a large effect on the total mean intake of most nutrients [18]. Furthermore, a study from the UK investigated alcohol consumption on atypical/special occasions such as holidays and celebrations and observed that the alcohol intake was substantial on these occasions, and the intake on typical days alone seemed to be a poor proxy for the actual alcohol consumption [19].

What normal and unusual days for dietary intake are for patients undergoing treatment for a disease, such as cancer, is uncertain. For newly diagnosed breast cancer patients, several factors may influence the dietary intake, such as hospitalization [20], and adjuvant breast cancer treatment [21, 22], as well as psychological distress related to the diagnosis [23]. Furthermore, a larger percentage of patients have been classified as having an “inadequate diet” after compared to before chemotherapy [24]. However, contradictory results have been described and weight gain is common post-diagnostic throughout and after adjuvant breast cancer treatment [24–27]. Overall, there are indications that nutritional factors can affect the prognosis of breast cancer, but this area of research is still limited. In particular, knowledge about nutritional factors in the period after a breast cancer diagnosis is limited [28]. Examining normal and unusual days for dietary intake among women newly diagnosed with breast cancer can contribute to shedding light on the diet in this period.

The present study investigates the dietary intake in days characterized as “unusual” compared with “normal” days among women newly diagnosed with breast cancer stage I or II during the 12-month post-surgery period. Furthermore, we examine the dietary intake grouped by weekdays and weekend days, and over time. Finally, as a contribution to increasing the knowledge about the pre-coded food diary assessment method we explore the dietary intake provided in the free text fields of a food diary/record.

## Materials and methods

### Study population and design

Women (18–75 years) newly diagnosed with invasive breast cancer (stages I and II) were invited to take part in the present study at the Oslo University Hospital, Oslo, St Olav University Hospital, Trondheim, and Vestre Viken, Drammen from September 2014 to October 2017. Breast cancer patients with known severe illnesses (i.e. dysregulated diabetes, severe heart disease), physical function making them unable to walk or not able to speak and write Norwegian were excluded. All eligible breast cancer patients who came to the outpatient clinic were invited and informed by a nurse and a trained oncologist before surgery or any treatment. The breast cancer patients followed standard breast cancer treatment [29] and were given general advice on having a diet in line with the Norwegian dietary guidelines [30].

A total of 457 breast cancer patients had dietary data available at a minimum of one time point, measured with the pre-coded food diary, and only one patient did not answer the question about the days being “normal” or “unusual” at all and was excluded from all analyses. Dietary data were available for 456, 399, and 380 breast cancer patients at 3 weeks, 6 months, and 12 months post-surgery. Reasons for not filling in the food diary were resection, recurrence of the disease, side effects of treatments, family settings (such as serious illness among family members), and time-consuming in a difficult time.

The total years of education, height, and weight were self-reported at diagnosis before any treatment, and body mass index was calculated (BMI, kg/m^2^).

### Dietary assessment, pre-coded food diary

All the participating breast cancer patients registered all the food that they consumed in a pre-coded food diary for 7 consecutive days 3 weeks post-surgery (before any other treatment), at 6 months post-surgery, and 12 months post-surgery (i.e. 3 × 7 days). None of the reported days included days where chemotherapy was administered. Each day of the food diary is 19 pages long, includes 310 questions about the intake of different food items, and has been previously described in detail [16]. Household units and a booklet [31] with photos of portion sizes were used to estimate the amounts consumed. On the front page of each day of the food diary, there are questions about age, day of the week, and date. In addition, there is a question: “Was today a normal day?”, where the participating breast cancer patients can answer “yes” or “no”. The intention was to capture “normal” or “unusual” days concerning the consumption of food and drinks. The question about normal/unusual days was answered on 98% (*n* = 8408) of the days. Over time, 452, 398, and 380 breast cancer patients answered the question about normal/unusual days on one or more days 3 weeks post-surgery, and at 6 and 12 months post-surgery, respectively. There are 25 free text fields throughout 1 day of the food diary, where food items not covered in the pre-coded part can be reported.

Trained personnel manually checked all the food diaries shortly after they were filled in and any inconsistencies or missing information was obtained by contacting the breast cancer patients by phone. The completed food diaries were scanned using Cardiff TeleForm program version 10.5.1 (Datascan Oslo, Norway), whereas the free text fields were manually coded. The scanned and coded data were imported into a dietary calculation software system (the KBS calculation software system) at the Department of Nutrition, Institute of Basic Medical Sciences, University of Oslo. The food database AE-14 was used for the computerization and calculations of the dietary data (for both the pre-coded part as well as the free text fields), which is an extended version of the official Norwegian food composition tables from 2014 and 2015 [32], supplemented with data from calculated recipes and other databases. Mixed dishes were split into their food items/ingredients before analysis.

The pre-coded food diary is developed and validated by the Department of Nutrition, Institute of Basic Medical Sciences, University of Oslo [33–36], also including breast cancer patients [16].

### Food categories and nutrients

Nutrients and food categories in focus were related to highlighted categories/nutrients in the world cancer research fund (WCRF)/American institute for cancer research (AICR): the continuous updating project (CUP) report for Breast Cancer Survivors; the CUP report for Breast Cancer Prevention; and the Cancer Prevention Recommendations [28, 37, 38]. Food categories in focus included fruit and berries that were all fresh/frozen (97% of consumed fruit/berries) or processed fruit and berries with > 50% fruit and berries (e.g. canned or jam) and vegetables including all fresh/frozen vegetables (90% of all vegetables), as well as canned and conserved vegetables. Furthermore, it included the category red and processed meat, which was made up of processed meat (66% of the meat), which includes all meats (red and white) that are cured, smoked, salted, fermented, or processed in any way to improve the taste or for preservation. This category also consisted of red meat including all (unprocessed) red meat such as pork, lamb, beef, veal, and mutton. The category dairy products were made up of milk products (80%), which not only included all milk, yoghurt, quark, and cream, but also cheese (20%), including all types of cheese. The category sweets, cakes, and snacks consisted of the following: sugar and sweets (28%); desserts (21%), which included cream-based desserts, puddings, and ice-cream; cakes (41%), which included yeast-based buns and similar, waffles, sweet biscuits and all sorts of other cakes; and snacks (10%), which included all salty snacks. In addition, the intake of total energy, carbohydrates, sugar, fiber, protein, alcohol, fat, and saturated fat was assessed.

For the intake reported in the free text fields of the food diary, the food categories that contributed the most energy in most of the breast cancer patients in the present study were calculated. From these food categories the following were selected (highlighted by the WCRF/AICR [28, 37, 38]): fruit and berries; vegetables; red and processed meat; dairy products; and sweets, cakes, and snacks.

### Tumor characteristics and breast cancer treatment

All breast cancer surgical specimens were histologically and immunohistochemically examined. The breast cancer tumors were classified according to invasive histological type [No Special Type (NST), lobular, and others], histological grade (1–3), and tumor diameter was measured both macro- and microscopically (mm). Lymph nodes were investigated to detect macro- or micro-metastases, using sentinel lymph node (SN) biopsy technique for identifying axillary metastases. The tumors were routinely examined by immunohistochemistry for the following markers: estrogen receptor (ER positive status as ≥ 1% ER-expressing tumor cells), progesterone receptor (PgR positive status as ≥ 10% PgR-expressing tumor cells), human epidermal growth factor receptor 2 (HER2), and tumor cell proliferation (Ki-67 hotspot index). HER2 immunohistochemistry equivocal cases (2 +) were examined using HER2 Dual SISH in situ hybridization to detect gene amplification. The ER negative, PgR negative, and HER2 negative tumors were aggregated to the molecular subtype triple negative breast cancer (TNBC). The expression (as percentage) of Ki-67-positive tumor cells was determined according to national and international guidelines [29, 39]. Further details about the assessment of tumor characteristics in the present study are described elsewhere [40].

All patients in the present study were treated according to national breast cancer treatment guidelines (nbcg.no) and underwent surgical removal of the tumour(s) with either breast-conserving treatment, mastectomy or mastectomy with primary reconstruction, and furthermore ipsilateral sentinel node biopsy or axillary dissection. Age, TNM classification (Tumour size, Nodal metastasis, distant Metastasis), and tumour biology (grade, hormone receptor status, HER2 receptor status, proliferation) guided systemic adjuvant treatment recommendations. The chemotherapy regimens used were either fluorouracil, epirubicine, and cyclophosphamide (FEC) every third week for 6 cycles, or 4 cycles of FEC followed by taxanes for 12 weeks (12 weekly paclitaxel or 4 docetaxel every third week). In 2015, treatment guidelines removed fluorouracil from the chemotherapy regimens and changed to 4 cycles of epirubicine and cyclophosphamide (EC). HER2 positive patients were treated with trastuzumab every third week for a year, with trastuzumab starting concomitantly with the taxanes. Patients who required chemotherapy started treatment 4–6 weeks after surgery [29]. Breast cancer patients treated with breast-conserving surgery without lymph node metastasis were offered adjuvant whole breast irradiation, and patients with lymph node metastasis were offered adjuvant irradiation to the breast/chest wall and locoregional lymph nodes. In hormone receptor positive disease, endocrine therapies were given with anti-estrogen/Tamoxifen in pre- and peri-menopausal women, and aromatase inhibitors in postmenopausal women. In addition, goserelin (LHRH agonist) were recommended in young women under the age of 35 years from 2015. Endocrine therapy (recommended treatment for 5–10 years) was indicated for most breast cancer patients with tumors expressing hormone receptors, except for patients with the smaller tumors with less aggressive biology. Postmenopausal women (including chemically and surgically induced postmenopausal status) were also offered treatment with bisphosphonates for 5 years [29].

### Statistical analyses

The hypotheses were defined and analyses planned before they were performed. Descriptive statistics of the participating breast cancer patients [age, education, weight, height, and Body mass index (BMI, kg/m^2^)] are presented as the mean [95% confidence interval (CI)], and categories of BMI (normal ≤ 25 kg/m^2^, overweight = 25–30 kg/m^2^, and obese ≥ 30 kg/m^2^), characteristics of the tumor and adjuvant treatment are presented by percentage.

The number (%) of normal and unusual days was calculated both in total and at each time point. Logistic mixed models were used to estimate the odds ratio (OR, 95% CI) of an unusual day 6- and 12 months post-surgery compared with 3 weeks post-surgery. Linear mixed models were used to estimate differences in food and nutrient intake between the normal and unusual days, over time, and between normal/unusual weekdays (Monday, Tuesday, Wednesday, and Thursday) and weekend days (Friday, Saturday, and Sunday). Estimated marginal means (95% CIs) of energy, macronutrients [in megajoule (MJ), grams (g), and as energy percent (*E%*) per day], and selected food groups (g/day), were calculated and plots of the estimated margins (95% CIs) were created to visualize the intake over time.

For dietary intake reported in the free text fields, the mean (95% CI) intake in g/day or MJ/day, the percentage contribution to total mean intake, and the number (%) of days in which any intake of a given nutrient/food group was reported were calculated. Logistic mixed models were used to estimate the OR (95% CI) of reporting intake of a given nutrient/food group in the free text fields overall and at 6 and 12 months post-surgery compared with 3 weeks post-surgery. In addition, the OR of having an intake of the selected nutrients/food groups on the normal versus unusual days was estimated using logistic mixed models.

Breast cancer patients were included in the analyses if they had dietary data available at a minimum of 1 day at one or more time points. More than 98% of all food diaries had all 7 days filled out, and none had fewer than 4 days. The models were fitted and account for repeated measures (random effects: patient ID and time) within the individual over time (7 repeated days × 3 time points). To avoid making assumptions about the structure of data that were too strong, an unstructured covariance matrix for the random effects was used. In a few situations, an independent covariance matrix was used to avoid numerical non-convergence during estimation of the models. Sensitivity analyses for dietary intake were performed by including only breast cancer patients with complete dietary data (7 repeated days × 3 time points, and answered the question about normal/unusual day), and the results were approximately the same as for all participating breast cancer patients. A significance criterion of *P* < 0.05 was used. However, most importantly, the statistically significant findings were considered according to the actual sizes and relevance. All statistical analyses were performed using the statistical software package Stata SE version 15.1 (StataCorp LLC, College Station, TX).

## Results

At study inclusion (pre-surgery), the participating breast cancer patients’ mean age was 55.5 years with a mean BMI at 25.6 kg/m^2^ (Table 1). In total, 73.1% of the breast cancer patients underwent breast-conserving surgery (BCT) and the breast tumors were on average 17.1 mm and 87.5% were ER positive. A total of 83.2% of the breast cancer patients underwent radiotherapy, 56.5% chemotherapy, and 60.0% endocrine therapy.

**Table 1:** Descriptive statistics of the women diagnosed with stage I or II breast cancer (*n* = 456)

Characteristics at diagnosis	%	Mean (95% CI)
Age at diagnosis, years		55.5 (54.6, 56.4)
Education, years		15.0 (14.7, 15.3)
Weight, kg		71.8 (70.6, 73.1)
Height, cm		167.4 (166.8, 168.0)
BMI, kg/m^2^		25.6 (25.2, 26.0)
BMI category		
< 25 normal	50.4	
25–30 overweight	34.0	
> 30 obese	15.6	
Tumor characteristics		
Invasive breast carcinoma NST	74.3	
Invasive lobular carcinoma	14.3	
Other	11.4	
Tumor diameter, mm		17.1 (16.2, 18.1)
Histologic grade		
1	25.8	
2	46.4	
3	27.8	
Lymph node positive	25.1	
ER positive	87.5	
PgR positive	67.2	
TNBC	7.3	
Ki-67 hot spot, %		30.6 (28.4, 32.8)
HER2 positive	15.2	
Treatment		
Breast-conserving surgery	73.1	
Mastectomy	26.9	
Chemotherapy	56.5	
Radiotherapy	83.2	
Endocrine therapy	60.0	

Numbers may vary due to missing information

*BMI* body mass index, *CI* confidence interval, *ER* estrogen receptor, *HER2* Human epidermal growth factor receptor 2, *NST* no special type, *PgR* progesterone receptor, *TNBC* Triple negative breast cancer

### The dietary intake on normal and unusual days during the 12 month post-surgery period

In total, 89% of the breast cancer patients reported at least one day as unusual out of the 21 recorded days (7 days × 3 time points) during the 12-month post-surgery period. Out of all the recording days (*n* = 8408), 26% were reported as unusual days (Table 2). Furthermore, 5 days were reported as unusual with no dietary intake. The odds of reporting an unusual day did not differ by BMI category (normal/overweight/obese) or age (under/over 55 years of age, details not shown). An 11% higher energy intake was observed on the days reported as unusual compared with the normal days (Table 3, *P* < 0.001). The intake of most macronutrients (g/day) was 5–10% higher, except for fiber which was 7% lower, on the unusual days than on the normal days. However, the intake of sugar was 26% higher and the intake of alcohol 126% higher on the unusual days and, expressed as *E%*, those were the only macronutrients that were higher on the unusual than on the normal days. The energy from sugar was 125 kJ/day (95% CI 102–148) higher, whereas the energy from alcohol was 325 kJ/day (95% CI 297–352) higher, explaining 15% and 40%, respectively, of the higher energy intake on the unusual days than normal days.

**Table 2:** Number (%) of normal/unusual days and OR of an unusual day compared to normal day among the participating breast cancer patients from 3 weeks post-surgery to 6 and 12 months post-surgery (*n* = 456)

	Total	Post-surgery		
		3 weeks	6 months	12 months
Normal days, *n* (%)	6184 (74)	2103 (68)	2057 (76)	2024 (78)
Unusual days, *n* (*%*)	2224 (26)	975 (32)	663 (24)	586 (22)
Total days, *n*	8408	3078	2 720	2610
OR (95% CI) of an unusual day		1	0.7 (0.6, 0.8)*	0.5 (0.4, 0.7)*

Post-surgery, at 3 weeks post-surgery, 6 months post-surgery, 12 months post-surgery

*CI* confidence interval

*Odds ratio (OR) in a logistic mixed model; significance *P* < 0.001

**Table 3:** Estimated mean dietary intake per day among the participating breast cancer patients (*n* = 456) during the 12-month post-surgery period on normal and unusual days

Dietary intake per day	Normal day (*n* = 6184)	Unusual day (*n* = 2224)	Difference^b^
	Mean (95% CI)^a^	Mean (95% CI)^a^	Mean (95% CI)
Energy, MJ	**7.4** (7.3, 7.6)	**8.2** (8.1, 8.4)	**0.8** (0.7, 0.9)*
Carbohydrate, g	**172** (167, 176)	**183** (178, 187)	**11** (8.1, 14)*
*E%*	**40** (39, 40)	**39** (38, 39)	− **1.2** (− 1.6, − 0.8)*
Sugar, g	**29** (28, 31)	**37** (35, 39)	**7.4** (6.0, 8.7)*
*E%*	**6.5** (6.2, 6.8)	**7.4** (7.1, 7.7)	**0.9** (0.7, 1.2)*
Fiber, g	**19** (19, 20)	**18** (17, 18)	− **1.4** (− 1.7, − 1.0)*
Protein, g	**75** (74, 77)	**79** (78, 81)	**4.1** (2.7, 5.5)*
*E%*	**18** (17, 18)	**17** (16, 17)	− **0.9** (− 1.1, − 0.7)*
Alcohol, g	**8.7** (7.6, 9.8)	**20** (19, 21)	**11** (10, 12)*
*E%*	**3.2** (2.9, 3.6)	**6.5** (6.0, 6.9)	**3.2** (2.9, 3.5)*
Fat, g	**76** (74, 78)	**83** (80, 85)	**6.3** (4.7, 8.0)*
*E%*	**37** (37, 38)	**37** (36, 37)	− **0.8** (− 1.2, − 0.4)*
SFA, g	**30** (29, 31)	**33** (32, 34)	**2.7** (2.0, 3.4)*
*E%*	**15** (14, 15)	**15** (14, 15)	− **0.1** (− 0.3, 0.1)
Fruit and berries, g	**166** (156, 176)	**138** (126, 149)	− **28** (− 36, − 21)*
Vegetables, g	**155** (149, 161)	**139** (132, 146)	− **16** (− 22, − 10)*
Red and processed meat, g	**69** (66, 73)	**78** (74, 83)	**9.1** (4.7, 13)*
Dairy products, g	**210** (197, 223)	**192** (178, 206)	− **18** (− 25, − 10)*
Sweets, cakes, and snacks, g	**69** (65, 73)	**95** (90, 99)	**26** (22, 30)*

*CI* confidence interval, *E%* percentage energy from nutrient, *MJ* megajoule, *SFA* saturated fatty acids

^a^Estimated mean (95% CI) intake in a linear mixed model

^b^Difference (95% CI) between intake on normal and unusual days in a linear mixed model

*significance *P* < 0.001

Intake of fruit and berries, vegetables, and dairy products was between 9 and 17% lower on the unusual days than the normal days (*P* < 0.001, Table 3). The lower intake of dairy products was mostly due to a lower intake of milk products (80% of the dairy products), whereas the intake of cheese was similar on the normal and the unusual days. The intake of red and processed meat, and sweets, cakes, and snacks was 13% and 38% higher on unusual days than the normal days, respectively.

Of note, less than 5% of all days were registered during the holiday periods of Christmas (between 24 December and 1 January), Easter (from the Palm Sunday weekend before Easter until the second Easter day), and the whole of July (summer holiday month in Norway). In total, these holiday periods typically account for 14% of the days over a year. Apart from these periods, where the number of scheduled study visits was reduced, the study visits were distributed all year round over 3 years.

### Differences between the normal and the unusual days over time

**Fig. 1:**
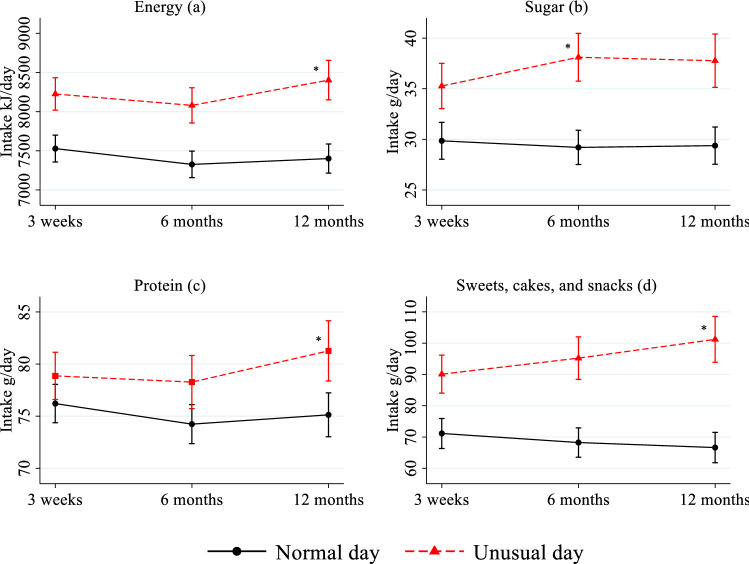
Estimated mean (95% CI) dietary intake per day and differences among the participating breast cancer patients (*n* = 456) in intake of energy (**a**), sugar (**b**), protein (**c**), and sweets, cakes, and snacks (**d)** over time on the normal and unusual days. *CI*, confidence interval. Post-surgery, at 3 weeks post-surgery, 6 months post-surgery, 12 months post-surgery. *Different from the difference 3 weeks post-surgery; significance *P* < 0.05

Over time, fewer breast cancer patients reported ≥ 1 unusual days: 75% at 3 weeks post-surgery, 67% at 6 months post-surgery, and 64% at 12 months post-surgery. Also, fewer total days were reported as unusual (Table 2). However, the differences reported in dietary intake between the normal and the unusual days mostly did not change over the 12 months post-surgery, except for an increased difference in the intake of energy, sugar in grams and *E%*, protein in grams, and sweets, cakes, and snacks (*P* < 0.05, Fig. 1). The mean intake of energy (Fig. 1a) was 7.5 MJ (95% CI 7.4–7.7) on the normal days and 8.2 MJ (95% CI 8.0–8.4) on the unusual days 3 weeks post-surgery. At 12 months post-surgery, the difference was larger (*P* < 0.05) with a mean energy intake of 7.4 MJ (95% CI 7.2–7.6) and 8.4 MJ (95% CI 8.2–8.7) for the normal and the unusual days, respectively.

The sugar intake on the normal days was 30 g/day (95% CI 28–32) 3 weeks post-surgery, and 29 g/day (95% 28–31) 6 months post-surgery (Fig. 1b). The intake on the unusual days was 35 g/day (95% CI 33–38), 3 weeks post-surgery and significantly higher (*P* < 0.05) at 6 months post-surgery, with 38 g/day (95% CI 36–40). A similar pattern was seen for *E%* from sugar (details not shown).

The mean protein intake 3 weeks post-surgery was 76 g/day (95% CI 74–78) on the normal days and 79 g/day (95% CI 77–81) on the unusual days (Fig. 1c). The difference was greater (*P* < 0.05) at 12 months post-surgery, demonstrated by an intake of 75 g/day (95% CI 73–77) on the normal days and 81 g/day (95% CI 78–84) on the unusual days.

The intake of sweets, cakes, and snacks on the normal days was 71 g/day (95% CI 66–76) 3 weeks post-surgery, and 67 g/day (95% CI 62–72) 12 months post-surgery (Fig. 1d). The intake on the unusual days was 90 g/day (95% CI 84–96) 3 weeks post-surgery, and higher (*P* < 0.05) at 12 months post-surgery at 101 g/day (95% CI 94–109).

### Normal and unusual weekdays and weekend days during the 12-month post-surgery period

The odds of reporting an unusual day during the 12-month post-surgery period was slightly higher for the weekend days than for the weekdays (OR 1.4, 95% CI 1.3–1.6, *P* < 0.001), with 24% of all weekdays and 30% of all weekend days reported as unusual days, respectively. The dietary intake of energy and macronutrients was higher (*P* < 0.05), apart from the intake of fiber, which was lower, on the unusual weekdays and weekend days than the respective normal days (Table 4). However, when expressed as *E%*, only the intake of sugar and alcohol was higher on the unusual weekdays and weekend days than the normal weekdays and weekend days, respectively. Specifically, Saturday was the day of the week with the highest intake of sugar and alcohol for both normal and unusual days, and Monday and Tuesday the days with the lowest intake on normal and unusual days, respectively. During the weekdays, alcohol was consumed on 20% of the normal days and 40% of the unusual days, whereas for the weekend days, it was consumed on 48% and 60% of the days, respectively. For the food groups, the intake of fruit and berries, vegetables, and dairy products was lower (*P* < 0.05) on the unusual weekdays and weekend days compared with the normal weekdays and weekend days, respectively, whereas the intake of red and processed meat, and sweets, cakes, and snacks was higher (*P* < 0.05). Sweets, cakes, and snacks were consumed on 76% of the normal weekdays and 81% of the unusual weekdays, whereas they were consumed on 86% of the normal weekend days and 87% of the unusual weekend days.

**Table 4:** The dietary intake per day among the participating breast cancer patients (*n* = 456) on the normal and unusual days by weekdays and weekend days during the 12-month post-surgery period

Dietary intake	Normal days		Unusual days	
	Week^a^ (*n* = 3632)	Weekend^b^ (*n* = 2552)	Week^a^ (*n* = 1 152)	Weekend^b^ (*n* = 1072)
	Mean (95% CI)^c^	Mean (95% CI)^c^	Mean (95% CI)^c^	Mean (95% CI)^c^
Energy, MJ	**7.0** (6.9, 7.2)	**8.0** (7.9, 8.2)	**7.7** (7.5, 7.9)*	**8.8** (8.6, 8.9)^†^
Carbohydrate, g	**165** (161, 169)	**181** (177, 186)	**176** (171, 181)*	**190** (184, 195)^†^
*E%*	**41** (40, 41)	**39** (38, 39)	**39** (39, 40)*	**38** (37, 38)^†^
Sugar, g	**26** (24, 27)	**35** (33, 36)	**33** (32, 35)*	**40** (38, 42)^†^
*E%*	**6.1** (5.8, 6.4)	**7.1** (6.8, 7.4)	**7.1** (6.7, 7.5)*	**7.7** (7.3, 8.1)^†^
Fiber, g	**19** (19, 20)	**19** (18, 20)	**18** (17, 19)*	**18** (17, 18)^†^
Protein, g	**73** (71, 75)	**79** (77, 81)	**77** (75, 79)*	**82** (80, 84)^†^
*E%*	**18** (18, 18)	**17** (17, 17)	**17** (17, 17)*	**16** (16, 17)^†^
Alcohol, g	**4.6** (3.4, 5.7)	**15** (14, 16)	**14** (13, 15)*	**26** (25, 27)^†^
*E%*	**1.9** (1.5, 2.3)	**5.2** (4.8, 5.6)	**4.9** (4.4, 5.4)*	**8.0** (7.5, 8.6)^†^
Fat, g	**72** (71, 74)	**82** (80, 84)	**78** (75, 80)*	**87** (85, 90)^†^
*E%*	**37** (37, 38)	**37** (37, 38)	**37** (36, 37)*	**36** (36, 37)^†^
SFA, g	**28** (27, 29)	**33** (32, 33)	**31** (30, 32)*	**35** (34, 36)^†^
*E%*	**15** (14, 15)	**15** (14, 15)	**15** (14, 15)	**15** (14, 15)
Fruit and berries, g	**173** (162, 183)	**156** (144, 167)	**146** (133, 158)*	**130** (116, 142)^†^
Vegetables, g	**156** (150, 162)	**153** (147, 160)	**137** (129, 146)*	**140** (132, 149)^†^
Red and processed meat, g	**63** (59, 67)	**78** (74, 82)	**70** (65, 76)*	**86** (81, 92)^†^
Dairy products, g	**214** (201, 228)	**203** (190, 217)	**196** (181, 211)*	**189** (173, 204)^†^
Sweets, cakes, and snacks, g	**56** (52, 60)	**87** (83, 91)	**79** (74, 85)*	**110** (105, 116)^†^

*CI* confidence interval, *E%* percentage energy from nutrient, *MJ* megajoule, *SFA* saturated fatty acids

*Significantly different from normal weekdays; significance *P* < 0.05

^†^Significantly different from normal weekend days; significance *P* < 0.05

^a^Weekdays: Monday, Tuesday, Wednesday, Thursday

^b^Weekend days: Friday, Saturday, Sunday

^c^Estimated mean intake in a linear mixed model

### Dietary intake reported in the free text fields

In total, 99% of the breast cancer patients used the free text fields in the pre-coded food diaries to report any additional dietary intake, ranging from 98% 3 weeks post-surgery to 91% 12 months post-surgery.

For the intake of energy, the mean contribution from the free text fields during the 12-month post-surgery period was 1.1 MJ/day (Table 5) and, in 74% of all recording days, there was an intake of energy in the free text fields. Furthermore, the odds of having an intake of energy or any macronutrients in the free text fields were lower 6 and 12 months post-surgery than 3 weeks post-surgery, except for the intake of alcohol.

**Table 5:** Total dietary intake per day among the participating breast cancer patients (*n* = 456) and intake in the free text fields per day during the 12-month post-surgery period

Dietary intake	Total intake	Free text fields		
	Mean (95% CI)	Mean (95% CI)	Percentage of total^a^	Percentage (*n*) days^b^
Energy, MJ	**7.6** (7.6, 7.7)	**1.1** (1.1, 1.1)	**14**	**74** (6 333)
Carbohydrate, g	**175** (173, 176)	**20** (19, 20)	**11**	**70** (6 069)
Sugar, g	**31** (31, 32)	**3.8** (3.6, 4.1)	**13**	**34** (2 895)
Fiber, g	**19** (19, 19)	**3.4** (3.3, 3.5)	**16**	**67** (5 731)
Protein, g	**76** (76, 77)	**11** (11, 11)	**14**	**71** (6 124)
Alcohol, g	**12** (11, 12)	**0.5** (0.4, 0.6)	**4.9**	**2.9** (248)
Fat, g	**78** (77, 79)	**14** (13, 14)	**17**	**70** (6 042)
SFA, g	**31** (30, 31)	**4.3** (4.1, 4.5)	**13**	**65** (5 597)
Fruit and berries, g	**159** (156, 163)	**22** (21, 23)	**20**	**27** (2 290)
Vegetables, g	**151** (149, 154)	**36** (34, 37)	**23**	**37** (3 212)
Red and processed meat, g	**71** (70, 73)	**8.3** (7.6, 9.0)	**10**	**10** (896)
Dairy products, g	**206** (202, 210)	**22** (20, 23)	**11**	**22** (1 936)
Sweets, cakes, and snacks, g	**76** (74, 78)	**9.9** (9.2, 10)	**14**	**21** (1 770)

Free text fields: sections in the pre-coded food diary for writing food consumed

*CI* confidence interval, *MJ* megajoule, *SFA* saturated fatty acids

^a^The mean percentage of free text fields contributing to total intake

^b^The percentage (*n*) days with a reported intake

The mean reported intake of fruit and berries in the free text fields during the 12-month post-surgery period was 22 g/day (Table 5), where blueberries (3.8 g/day, 95% CI 3.3–4.4), mango (3.7 g/day, 95% CI 2.9–4.4), and plums (2.9 g/day, 95% CI 2.0–3.7) were reported the most. For vegetables, the free text fields contributed 36 g/day, with avocadoes (6.3 g/day, 95% CI 5.4–7.2) and tomatoes (5.2 g/day, 95% CI 4.5–5.9) being the vegetables most reported. The mean reported intake of dairy products in the free text fields was 22 g/day, and plain yoghurt (4.5 g/day, 95% CI 3.4–5.6), cottage cheese (2.9 g/day, 95% CI 2.1–3.6), and skyr/quark (2.5 g/day, 95% CI 1.6–3.3) were reported the most.

The odds of reporting any intake of fruit and berries, and dairy products in the free text fields was lower at 12 months post-surgery than 3 weeks post-surgery, and for vegetables lower at both 6 and 12 months post-surgery compared to 3 weeks post-surgery.

### The free text fields on normal and unusual days during the 12-month post-surgery period

The mean energy intake from the free text fields was 1052 kJ (95% CI 976–1128) on the normal days and 15% (158 kJ, 95% CI 96–220, *P* < 0.001) higher on the unusual days at 1210 kJ (95% CI 1124–1296) during the 12-month post-surgery period. However, the odds of having an intake of energy or macronutrients in the free text fields did not differ between the normal and unusual days. One exception was a higher odds of having an intake of sugar on the unusual days (OR 1.6, 95% CI 1.4–1.8, *P* < 0.001), where 31% and 41% of the normal and unusual days had an intake in the free text fields, respectively. Also, there were higher odds of having an intake of alcohol (OR 3.2, 95% CI 2.4–4.3, *P* < 0.001) on the unusual days than on the normal days, with an intake in the free text fields on 2% and 6% of the normal and unusual days, respectively.

For the food groups, there were no differences in the odds of having an intake of fruit and berries, vegetables, and dairy products in the free text fields between the normal and the unusual days over the 12-month post-surgery period. However, there were higher odds of reporting intake of red and processed meat (OR 1.4, 95% CI 1.2–1.7, *P* < 0.001), and sweets, cakes, and snacks (OR 1.5, 95% CI 1.3–1.8, *P* < 0.001) in the free text fields on the unusual days compared with the normal days. For red and processed meat, any intake in the free text fields was reported on 10% of the normal days and 13% of the unusual days, and for sweets, cakes, and snacks it was reported on 19% of the normal days and 25% of the unusual days.

## Discussion

In the present study among female breast cancer patients, we found that 26% of all days were reported as unusual days, and these days were characterized by a less healthy dietary pattern than the normal days, in particular for the intake of alcohol. Normal and unusual days, grouped by weekdays or weekend days and over time, showed the same pattern as total days. Overall, 99% of the patients used the free text fields of the food diary and this additional reporting contributed substantially to total dietary intake per day. Mostly, the free text fields were used equally on the normal and unusual days.

To the best of our knowledge, we are the first to study normal and unusual days for dietary intake, and to report on dietary intake provided in free text fields in a food diary/record during the 12-month period after a breast cancer diagnosis. Thus, our results extend previous results, but are also supported in parts by others, such as in a subsample of the Norwegian women and cancer cohort (NOWAC), where 24% and 34% of the days were reported as special days for dietary data collected by 24-h recalls by telephone and face-to-face, respectively [41]. In the UK’s National Diet and Nutrition Survey (8 006 participants with complete dietary data from both sexes, aged 13–96 years) 64% of the participants reported ≥ 1 day with unusual intake out of 4 recording days [42]. In line with this, we found that the percentage of breast cancer patients reporting ≥ 1 unusual day out of 7 days ranged from 75% 3 weeks post-surgery to 64% at 12 months post-surgery. Interestingly, among 1090 healthy postmenopausal women from the Women’s Trial Feasibility Study in Minority Populations in the US, only 16% of the participants had food records with ≥ 1 atypical days out of 4 recording days [18]. The discrepancy is substantial, even when taking into account that the comparison is between 7 and 4 days of diet registration. Nevertheless, the total percentage of unusual days (26%) observed in our study was about the same (27%) as observed in a population-based Norwegian dietary survey (Norkost 3). However, the study population in Norkost 3 was men and women from the general Norwegian population, and a different dietary assessment method was used (2 × 24-h recalls) [43]. Importantly, all the mentioned studies [18, 41–43] are in a general or a healthy population, in contrast to the present study that is among patients undergoing adjuvant breast cancer treatment.

Of note, 15% of the difference in energy intake between normal and unusual days could be explained by the 26% higher intake of sugar on the unusual days, and as much as 40% of the difference in energy intake could be explained by the 126% higher alcohol intake on the unusual days compared to the normal days. Moreover, the dietary intake on weekend days was less healthy than on weekdays, and we observed, in particular, a higher intake of total energy, alcohol, sugar and sweets, cakes, and snacks, which is similar to the results from other studies [5, 7, 8]. The odds of reporting an unusual day was higher for a weekend day compared with a weekday. A similar pattern was seen in the Women’s Trial Feasibility Study in Minority Populations, where days reported with more-than-usual food intake were most likely to occur on Fridays and Sundays and least likely to occur on Mondays when studying food records with fewer than 4 atypical days [18]. Even if most of the days in the present study were reported as normal weekdays (43%), with a low intake of alcohol, the intake of alcohol was high on normal weekend days and unusual weekdays, with the highest amount on unusual weekend days; together these days accounted for 57% of all days. These results are comparable with others, where the weekly alcohol consumption was the greatest on Fridays and Saturdays and the intake peaked on celebratory days [44].

Alcohol intake may increase the estrogen level [13], and be risk factor for breast cancer [37]. Furthermore, binge drinking and a high weekend consumption of alcohol have, in a few studies, been associated with an additional risk of breast cancer [45–47]. When it comes to alcohol and breast cancer recurrence and mortality, less is known [28], although alcohol consumption has been associated with an increased risk of breast cancer recurrence [48]. In general, the breast cancer patients in the present study were informed at study visits to follow the national and international guidelines regarding alcohol intake (included in the study period) and that they limit their consumption of alcohol to < 10 g/day or 7 units of alcohol/week. It is possible that ticking off the day as ‘unusual’ for some participants was a way of justifying reporting intake of alcoholic beverages and less healthy food items such as sweets, cakes, and snacks. However, it is unknown whether the reported intake would have been different if the question about normal/unusual days had not been asked. Nevertheless, there is a possibility of social desirability bias and/or reactivity due to filling in the pre-coded food diary and consequently underreporting the alcohol intake and less healthy food items [4, 49].

The meaning behind the question about normal/unusual day was intended to be about dietary intake, but unfortunately, it is phrased in a way that does not distinguish between a normal/unusual day in general and a normal/unusual day concerning dietary intake. Most probably, unusual days both in general and concerning dietary intake may have been captured. In the UK’s National Diet and Nutrition Survey 9% of the participants reported ≥ 1 days with unusual intake due to illness or medical reasons, whereas 51% of participants reported unusual intake day(s) with reasons such as ‘working’, ‘at friends’, ‘weekend’, and ‘with family’, reasons that were considered as part of the normal day-to-day variability in consumption [42]. The setting of the present study was women diagnosed with breast cancer stage I or II during a period of 12 months post-surgery and, therefore, undergoing adjuvant breast cancer treatment. However, dietary intake on the specific days of receiving chemotherapy was not collected in the present study, but would have been of interest. Thus, the unusual days could have been days with clinical visits at the hospital, or days with side effects from the treatment or a high level of distress related to the disease, as well as special occasions and settings that people in general experience during a 12-month period. In the present study, the typical unusual day is a day with a higher intake of most nutrients. Apparently, the unusual days with a lower dietary intake are too few or not so low as to counteract the higher-intake days. Only 5 days for the whole population were reported as unusual days with no dietary intake at all. Of note, is the fact that breast cancer patients may experience weight gain after being diagnosed with breast cancer [50, 51].

In the present study, fewer days were reported as unusual over time. although there were minor differences in the consumption of nutrients/food groups on the normal and unusual days over time. Previously, we have demonstrated that overall the breast cancer patients did mostly not change their diet during the 12-month post-surgery period [17]. Nevertheless, here we observe a small, statistically significant increase in the intake of energy and sweets, cakes, and snacks on the days characterized as unusual and a tendency toward a lower intake on the normal days over time. Together it seems that there is a trend toward a higher level for reporting a day as unusual over time, or possibly there are more unusual days with a low energy intake 3 weeks post-surgery than 12 months post-surgery. We have also previously demonstrated among these patients that they did not change their BMI during the same 12-month post-surgery period [17]. The mean unusual day represents a day with a higher energy intake and a less healthy dietary pattern. This emphasizes that this group of cancer patients do not need dietary advice on how to reduce their risk of malnutrition or weight loss in general, but rather focus on a healthy diet with more vegetables and particularly a lower intake of alcoholic beverages as is recommended to the general population [30]. The dietary focus should also be on the usual intake over time and the effect of unusual days as well as weekend days on the total diet.

In the present study, the free text fields are substantial contributors to total energy intake as well as the intake of several food groups and specific food items, especially fruit, berries, and vegetables. The free text fields are also important for reporting mixed dishes that are not mentioned in the pre-coded food diary. As an example, tomatoes were one of the most reported vegetables (in g/day) in the free text fields and this likely comes from mostly mixed dishes because there are two questions in the food diary about tomatoes, in particular, both a general question about sliced tomatoes and one about their use on bread.

Our findings demonstrate the benefits of using the free text fields and points at food groups/items that are not well captured in the pre-coded food diary. If only the pre-coded part of the food diary is used for assessing dietary intake and not the free text fields, it should be taken into account and discussed whether the dietary intake might be underreported, especially concerning the intake of total energy, fruit, berries, and vegetables. However, the benefits of using the free text fields are dependent on this specific pre-coded food diary (the instrument) and to what degree the food diary fits the purpose of the study and the study population it is intended to be used in. Furthermore, reporting in the free text fields may act as a suggestion to add new food items when updating the pre-coded part of the food diary such as blueberries, avocado, and plain yoghurt. Over time, the free text fields were used less which may indicate that using them is a bit tiring, although it is also possible that the breast cancer patients became more familiar with the pre-coded food diary and better at reporting what they ate in the pre-coded part. The reported intake in the free text fields on unusual and normal days had the same pattern as total intake on the respective days, meaning free text fields contributed to the less healthy dietary intake observed on the unusual days compared to the normal days.

The strengths of the present study include that the breast cancer patients met the same trained personnel throughout the study and that they were contacted if there was missing information or inconsistencies in the food diary. Moreover, a strength is the large number of early diagnosed breast cancer patients with the many repeated measures of dietary intake (7 days × 3; 21 days per person), with the possibility of examining the dietary intake on normal/unusual days, but also whether it changed from 3 weeks post-surgery to 6 and 12 months post-surgery. Nevertheless, there are also some limitations: the dietary intake and the definition of a normal and an unusual day was self-reported. From the validation of the pre-coded food diary, we experienced that there may be more uncertainty associated with the dietary assessment 3 weeks post-surgery than at 6 and 12 months post-surgery [16]. Patients that lived within the geographical areas of the three hospitals involved in the trial and who fulfilled the inclusion criteria were asked to participate. However, some potential participants might have been missed out as they were asked to participate before surgery and the final tumour diagnosis was set. Last, no adjustments were made for multiple testing even if many statistical tests were performed. However, the clinical relevance of the actual sizes and differences is most essential.

## Conclusion

In the present study among female breast cancer patients during the 12 month post-surgery period, days regarded as unusual were important drivers of total dietary intake and these days were less healthy than normal days, including a higher reported intake of alcohol. The same pattern was seen for normal and unusual days grouped by weekdays and weekend days, and over time. These findings emphasize the importance of including not only unusual days, but also weekend days when assessing dietary intake and the effect of these days on usual intake, in particular for the intake of alcohol. Furthermore, the free text fields in the pre-coded food diary contributed substantially to total dietary intake.

## Data Availability

The datasets used and analysed during the current study are not publicly available due to containing information that could compromise research participant privacy/consent but are available from the corresponding author on reasonable request.
